# Comparative Genomic Sequencing and Pathogenic Properties of Equine Herpesvirus 1 KyA and RacL11

**DOI:** 10.3389/fvets.2017.00211

**Published:** 2017-12-11

**Authors:** Akhalesh K. Shakya, Dennis J. O’Callaghan, Seong K. Kim

**Affiliations:** ^1^Department of Microbiology and Immunology, Center for Molecular and Tumor Virology, Louisiana State University Health Sciences Center, Shreveport, LA, United States

**Keywords:** equine herpesvirus 1, Kentucky A, attenuated virus, RacL11, Ab4, whole-genome sequencing, growth kinetics, pathogenesis

## Abstract

Equine herpesvirus 1 (EHV-1) is a major pathogen affecting equines worldwide. The virus causes respiratory disease, abortion, and, in some cases, neurological disease. EHV-1 Kentucky A (KyA) is attenuated in the mouse and equine, whereas wild-type pathogenic strain RacL11 induces severe inflammatory infiltration of the lung, causing infected mice to succumb. The complete DNA sequencing of the KyA genome revealed that genes UL17 (ORF17), US6 (ORF73; gI), US7 (ORF74; gE), and US8 (ORF75; 10 K) are deleted as compared to the RacL11 and Ab4 genomes. In-frame deletions in the US1 (ORF68), US4 (ORF71; gp2), and UL63 (ORF63; EICP0) genes and point mutations in 14 different open reading frames (ORFs) were detected in the KyA genome. Interestingly, UL1 (ORF1) and UL2 (ORF2) were deleted in both KyA and RacL11. Our previous studies showed that EHV-1 glycoproteins gI, gE, and full-length gp2 contribute to the pathogenesis of the RacL11 strain. The confirmation of these gene deletions in KyA suggests their contribution to the attenuation of this virus. The growth kinetics results revealed that KyA replicates to high titers in cell culture as compared to RacL11 and Ab4, indicating that the above genomic deletions and mutations in KyA do not have an inhibitory effect on KyA replication in cells of mouse, rabbit, equine, or human origin. Studies of EHV-1 pathogenesis in CBA mice showed that KyA is attenuated whereas mice infected with RacL11 succumbed by 3–6 days post-infection, which is consistent with our previous results.

## Introduction

Equine herpesvirus 1 (EHV-1) is a common pathogen of horses worldwide. EHV-1 is a member of the *Varicellovirus* genus within the subfamily *Alphaherpesvirinae* (1, 2) Infection causes upper respiratory tract disease, abortion in pregnant mares, neonatal death, and fatal myeloencephalopathy (3–5). The role of viral proteins ORF30 and glycoprotein D in the pathogenicity of myeloencephalopathy was reported recently as these two viral proteins are responsible for modulating host immune response (6). One of the main causes of abortion and neurological disease in horses is thrombosis in placental and spinal cord vessels. The findings of Stokol et al. (7) suggested that activation of platelets is a feature of EHV-1 pathogenicity and could be a factor leading to thrombosis. Important in EHV-1 pathogenicity is its ability to infect lymphoid tissue of the upper respiratory tract and spread to monocytes that mediate viremia (8, 9). By this means, the virus can reach the vasculature of the uterus and the central nervous system, resulting in severe clinical outcome (9, 10).

Several vaccines are in current use to prevent the most severe threat of EHV-1 infection. However, the currently available vaccines do not confer long-term protection against the severe manifestations of EHV-1 disease, such as fatal myeloencephalopathy. Therefore, there is an urgent need to develop vaccines that provide long-term protection. A better understanding of EHV-1 pathogenesis in relation to virus genomic sequences and specific alterations in the genome will help in the design of effective vaccines to reduce mortality and morbidity.

Equine herpesvirus 1 RacL11, isolated from an aborted foal (11), causes a severe infection of mice, resulting in significant weight loss, lethargy, ruffled fur, inflammation of the lungs, and a high mortality rate (12–14). The Ab4 strain of EHV-1 was isolated from a quadriplegic gelding (15), and it has been reported that deletion of gene US4 (ORF71) in the Ab4 genome resulted in attenuation. The gene 71-deleted virus mutant was evaluated as an experimental vaccine and was found to confer protection against pulmonary disease in mice after challenge with wild-type (wt) EHV-1 (16). The EHV-1 strain Kentucky A (KyA), a candidate vaccine strain, is non-pathogenic for both mice and horses after serial passage in mouse fibroblast L-M cells (17–20). The repeated passage in cells other than those of the natural host has resulted in the accumulation of genomic alterations of the KyA chromosome, including deletion of several genes or portions of open reading frames (ORFs).

The structure of the EHV-1 genome was first elucidated by detailed restriction enzyme mapping (21) and was confirmed by electron microscopic analyses (22). The EHV-1 genome comprises a unique long (U_L_) region and a short (S) region that consists of a unique segment (Us) bracketed by identical internal and terminal repeat, designated as IR and TR, respectively. The complete genomic sequence of the EHV-1 Ab4 was reported (23), and found to be 150,223 bp in length and to contain 76 distinct ORFs (23, 24). Six identical ORFs are present in the internal and terminal repeats of the EHV-1 genome as regulatory genes IR2 (ORF77) (25, 26) and IR3 (ORF78) (27–29) were identified in the KyA genome in addition to genes 64, 65, 66, and 67 as reported for Ab4 by Telford et al. (23).

Our previous DNA sequencing analyses of portions of the KyA genome revealed several gene deletions UL1 [ORF1], UL2 [ORF2], UL17 [ORF17], US6 [ORF73; gI], US7 [ORF74; gE], and US8 [ORF75] and in-frame deletions in UL63 (ORF63; EICP0) and US4 (ORF71; gp2) relative to the sequence of the wt Ab4 strain of EHV-1 (30–35). EHV-1 glycoproteins gI, gE, and gp2 contribute to the pathogenesis of the wt RacL11 strain (36–38). The deletion of ORF1/2 was found to impact clinical disease as ponies infected with the UL1/UL2 deleted virus had significantly shorter primary pyrexia, reduced nasal shedding, and a decrease in PBMC IL-8 as compared to ponies infected with wild-type virus (39). A single nucleotide polymorphism, N752 replaced by D752, in the gene encoding the viral DNA polymerase (ORF30) played a significant role in the magnitude and longer duration of viremia and enhancement of neurovirulence of naturally occurring EHV-1 strains (40).

The main objective of the present study was to determine the complete nucleotide sequence of the genomes of the attenuated EHV-1 KyA strain and the wt RacL11 strain and to compare these genomes in order to identify mutations associated with viral growth, cell tropism, and virulence. Comparison of the KyA and RacL11 genomes and additional comparison of these two genomes to that of wt Ab4 will help to identify viral gene(s) that are required for virulence and may trigger host cell gene expression, including innate immune responses. In addition, we determined the growth kinetics of the EHV-1 KyA, RacL11, and Ab4 strains in four different cell types, information that may eventually be helpful in the design of efficacious vaccines.

## Materials and Methods

### Cells and Viruses

Equine dermis (NBL6), rabbit kidney (RK-13), human epithelial kidney (HEK293), and mouse lung epithelial (MLE12) (ATCC: CRL-2110) cells were used for one-step growth experiments. NBL6, RK-13, and HEK293 cells were maintained in Eagle’s minimal medium supplemented with 100 units of penicillin/ml, 100 µg of streptomycin/ml, 1× non-essential amino acids, and 5% (or 10%) fetal bovine serum (FBS). MLE12 cells were maintained in HITES medium (ATCC) containing 2% FBS. Three EHV-1 strains KyA, RacL11, and Ab4 were used in the study. All three strains were grown in NBL6 cells.

### Mice

Three- to four-week-old female CBA mice were obtained from Harlan Sprague-Dawley (Indianapolis, IN, USA) and maintained in filter-topped cages at Animal Resource Facility of the Louisiana State University Health Science Center (LSUHSC), Shreveport. All mice were rested for 5 to 7 days prior to use.

### Infection and Assessment of Pathogenicity of EHV-1 Strains

To assess viral virulence, CBA mice (*n* = 5 per group) were anesthetized by isoflurane (Sigma Chemical Co., St. Louis, MO, USA) inhalation and were inoculated intranasally either with of 1.5 × 10^6^ plaque forming unit (PFU)/mouse of KyA, wild-type (wt) RacL11, or wt Ab4. Mice were monitored daily for observable clinical signs, such as abnormal respiration, crouching, ruffled fur, and huddling. All mice were also monitored for the loss of body weight. Animals exhibiting profound deterioration or a progressive decline in mobility were euthanized to prevent suffering. The significance of the percent survival to judge virulence was determined by GraphPad Prism software (GraphPad Software, Inc., La Jolla, CA, USA). Mice were sacrificed by CO_2_ inhalation, the lungs were removed at 2, 3, and 4 days post-infection (dpi), and virus titers were determined by plaque assay on NBL6 cells.

### Growth Kinetics in Cell Culture

NBL6, RK-13, HEK293, and MLE12 cells (8 × 10^5^) were seeded in six-well plates and infected at a multiplicity of infection (MOI) of 0.05. After 1 h of attachment at 37°C in 5% CO_2_, cells were washed with medium three times and overlaid with Dulbecco’s modification of Eagle’s medium (DMEM; Corning Inc., Manassas, VA, USA) containing 10% FBS. The plates were incubated in 5% CO_2_ at 37°C and harvested at 2, 12, 24, 36, and 48 h post-infection. Virus quantification was done by plaque assay on equine NBL6 cells. Briefly, freshly grown confluent NBL6 cells in 24 well plates (Corning, Inc., Manassas, VA, USA) were infected with a 10-fold serial dilution of each virus. After 1 h adsorption at 37°C, virus-infected cells were covered with medium containing 1.25% methylcellulose and incubated in 5% CO_2_ at 37°C. After 4 dpi, cells were fixed with 10% formalin and stained with 0.5% crystal violet to visualize the plaques. Growth kinetics results of KyA, RacL11, and Ab4 were statistically evaluated by analysis of variance (ANOVA) using GraphPad PrismTM version 5.0 software (GraphPad Software Inc., San Diego, CA, USA).

### Viral DNA Extraction and High-Throughput Sequencing

The genomes of KyA and RacL11 strains of EHV-1 were sequenced. EHV-1 KyA is attenuated in the mouse and equine (19, 20), whereas wild-type pathogenic strain RacL11 induces severe inflammatory infiltration of the lung causing infected mice to succumb (12). Total viral DNA was extracted from the supernatant of NBL6 cells infected with EHV-1 KyA or RacL11 using the QiAampMinElute virus spin kit (Qiagen, Valencia, CA, USA), according to the manufacturer’s instructions. Next-generation DNA sequencing was done on an Ion-torrent platform at PrimBio Research Institute, Philadelphia, PA, USA. The libraries of the EHV-1 strains were prepared using Ion Xpress Plus Fragment Library Kit (Thermo Fisher, South San Francisco, CA, USA). Briefly, 100 ng of virus DNA was enzymatically fragmented. Ion P1 adapter and barcodes were ligated to the fragmented DNA, and size selection was performed to obtain the fragments in the range of 200–350 bp. The libraries were further amplified with the Library Amplification Primer Mix. The final libraries were analyzed using the Agilent High Sensitivity DNA Analysis Kit (Agilent, Santa Clara, CA, USA). The library (26 pM) was templated and enriched using Ion PGM Hi-Q OT2 kit (Thermo Fisher). Sequencing was performed using an Ion Torrent PGM Sequencer and the Ion PGM Hi-Q Sequencing Kit (Thermo Fisher) with 850 sequencing flows. Point mutations were confirmed by Sanger sequencing at the DNA Core Facility of Iowa State University.

### Sequencing Data Analysis

DNA sequence assembly was done by AssemblerSPAdes (v5.0.0.0) and Mira Assembly. The complete genome sequences were assembled by reference sequence mapping with Mira Assembly using the genomic sequence of EHV-1 Ab4 strain (GenBank accession number AY665713). Alignment and comparison of DNA sequences were done by ClustlW2 and BioEdit sequence alignment editor. The phylogenetic tree was constructed to compare the genomes of KyA and RacL11 to those of 27 other equine herpesvirus strains of EHV-1, EHV-4, and EHV-9. The nucleotide sequences of the 27 other equine herpesvirus strains were obtained from the GenBank database. The phylogenetic tree was constructed by the neighbor-joining method using the Molecular Evolutionary Genetic Analysis (MEGA7) software (41) based on the nucleotide sequences. Node support was evaluated using 1,000 bootstrap replicates.

### Nucleotide Sequence Accession Numbers

The genomic sequences of KyA and RacL11 strains described in this work were deposited in GenBank under accession numbers MF975655 and MF975656, respectively.

## Results

**Figure 1:**
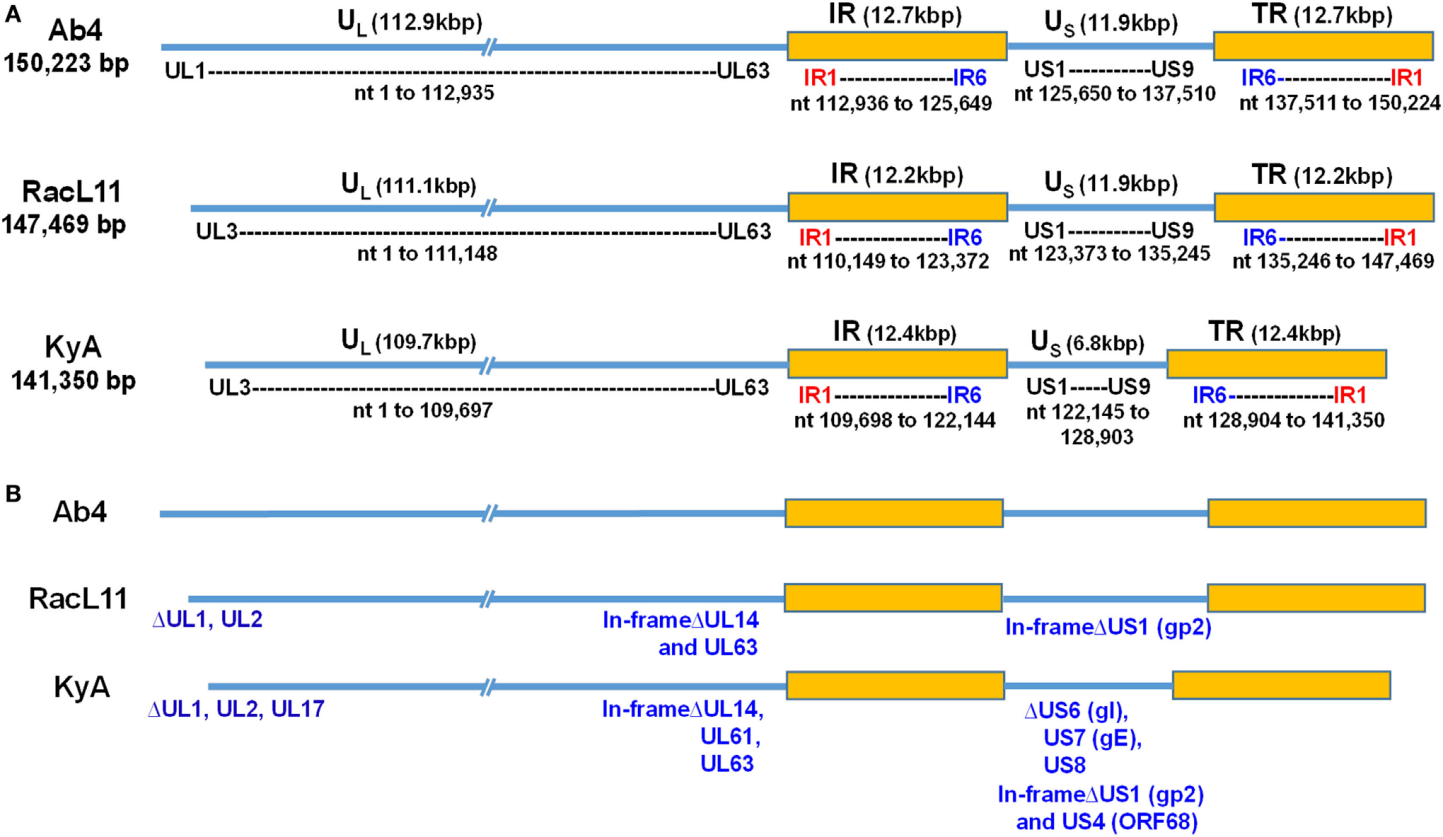
Schematic illustration of deletions in the genomes of equine herpesvirus 1 (EHV-1) Kentucky A (KyA), RacL11, and Ab4 strains. EHV-1 Ab4 sequences were used as the reference for mapping and annotation. **(A)** The two unique genomic segments, unique long (U_L_) and unique short (U_S_), and the internal and terminal repeat sequences (IR and TR, respectively) are shown. The sequencing results revealed that the genomes of KyA and RacL11 are 141,350 bp and 147,469 bp, respectively. nt, nucleotide. **(B)** Sequence analysis of the U_L_ region showed that the UL1 (ORF1) and UL2 (ORF2) genes are deleted in both KyA and RacL11. Deletion of UL17 was also observed in KyA. An identical in-frame deletion in UL63 (ORF63; EICP0) of both RacL11 and KyA was detected. The sequence analyses of the U_S_ segment showed that US6 (ORF73; gI), US7 (ORF74; gE), and US8 (ORF75; 10 K) genes were deleted in KyA. An in-frame deletion in the US1 (ORF68) and US4 (ORF71; gp2) genes was also detected in the KyA genome.

To elucidate the genetic relationships between the attenuated EHV-1 KyA and wild-type (wt) RacL11 and identify genomic mutations and alterations possibly associated with attenuation and virulence, whole genome sequencing was done. The EHV-1 Ab4 sequence was used as a reference for mapping and annotation. The sequencing results showed that the KyA genome is 141,350 bp and the RacL11 genome is 147,469 bp as compared to the wt EHV-1 Ab4 genome of 150,223 bp (Figure 1A). The comparison of sequences with Ab4 showed that the KyA and RacL11 genomes are 8,873 bp and 2,754 bp shorter, respectively. The G+C content of the KyA and RacL11 genomes is 57 and 56%, respectively. The G+C content of the Ab4 genome is 56.7% (23). A high G+C content is one of the features of the herpes simples virus (HSV) genomes as HSV-1 and HSV-2 have a G+C content of 68 and 70%, respectively, while the human genome has a G+C content 41% (42). Table 1 presents a comparison of the gene products of EHV-1 Ab4, RacL11, and KyA. The genomic sequencing data of this study and the results of a large number of publications on EHV-1 gene functions indicate that the wild-type EHV-1 chromosome harbors 63 ORFs in the UL region, 6 ORFs in each inverted repeat (IR and TR), and 9 ORFs in the Us segment. Although the function of many of the 78 distinct EHV-1 ORFs remains to be elucidated, studies indicate that most EHV-1 gene products characterized to data have functions similar to those of their homologs in the genome of HSV-1.

**Table 1:** Comparison of gene products of equine herpesvirus 1 (EHV-1) Ab4, RacL11, and Kentucky A (KyA).

Gene	Codons^a^			Function^b^	HSV-1
	Ab4	L11	KyA		
UL1 (ORF1)	202	–	–	MHC-1 downregulation and modulation of cytokine response	UL56
UL2 (ORF2)	205	–	–	Modulation of cytokine response	–
UL3 (ORF3)	257	257	257	Tegument protein; not essential for replication	–
UL4 (ORF4)	200	200	200	Inhibitory protein	UL55
UL5 (ORF5)	470	470	470	Post-translational regulator	UL54
UL6 (ORF6)	343	343	343	Glycoprotein K (gK)	UL53
UL7 (ORF7)	1081	1081	1081	Component of DNA Helicase/primase	UL52
UL8 (ORF8)	245	245	245	Tegument protein	UL51
UL9 (ORF9)	326	326	326	dUTPase	UL50
UL10 (ORF10)	100	100	100	Envelope protein	UL49A
UL11 (ORF11)	309	309	309	Involved in cell-to-cell spread	UL49
UL12 (ORF12)	476	476	476	Transactivator of IE gene	UL48
UL13 (ORF13)	871	871	871	Tegument protein	UL47
UL14 (ORF14)	747	626	626	Tegument protein	UL46
UL15 (ORF15)	219	219	219	Virion protein	UL45
UL16 (ORF16)	468	468	468	Glycoprotein C (gC); role in entry	UL44
UL17 (ORF17)	401	401	–	Membrane protein	UL43
UL18 (ORF18)	405	405	405	DNA polymerase processivity factor	UL42
UL19 (ORF19)	497	497	497	Host shutoff virion protein	UL41
UL20 (ORF20)	321	321	321	Ribonucleotide reductase, small subunit	UL40
UL21 (ORF21)	790	790	790	Ribonucleotide reductase, large subunit	UL39
UL22 (ORF22)	465	465	465	Capsid assembly protein	UL38
UL23 (ORF23)	1020	1020	1020	Tegument protein	UL37
UL24 (ORF24)	3421	3421	3421	Tegument protein; egress of virions through the cytoplasm	UL36
UL25 (ORF25)	119	119	119	Capsid protein	UL35
UL26 (ORF26)	275	275	275	Membrane-associated protein	UL34
UL27 (ORF27)	162	162	162	Role in DNA Packaging	UL33
UL28 (ORF28)	620	620	620	Cleavage/packaging	UL32
UL29 (ORF29)	326	326	326	Required for envelopment	UL31
UL30 (ORF30)	1220	1220	1220	DNA polymerase	UL30
UL31 (ORF31)	1209	1209	1209	DNA-binding protein; responsible for nuclear localization	UL29
UL32 (ORF32)	775	775	775	Cleavage/packaging	UL28
UL33 (ORF33)	980	980	980	Membrane glycoprotein B (gB); interacts with gH/gL and gD	UL27
UL34 (ORF34)	160	160	160	Essential for early step in virus egress	–
UL35 (ORF35)	646	646	646	Protease	UL26
UL35.5 (ORF35.5)	329	329	329	Capsid assembly protein	UL26.5
UL36 (ORF36)	587	587	587	Capsid protein involved in release of viral DNA	UL25
UL37 (ORF37)	272	272	272	Membrane-associated protein	UL24
UL38 (ORF38)	352	352	352	Thymidine kinase	UL23
UL39 (ORF39)	848	848	848	Glycoprotein H; essential for infectivity and fusion	UL22
UL40 (ORF40)	530	530	530	Tegument protein	UL21
UL41 (ORF41)	239	239	239	Membrane protein	UL20
UL42 (ORF42)	1376	1376	1376	VP5 major capsid protein	UL19
UL43 (ORF43)	314	314	314	VP23 capsid protein	UL18
UL44/47 (ORF44/47)	734	734	734	Tegument protein required for cleavage/packaging of DNA	UL15
UL45 (ORF45)	706	706	706	Capsid-associated required for cleavage/packaging	UL17
UL46 (ORF46)	370	370	370	Packaging	UL16
UL48 (ORF48)	317	317	317	Tegument protein	UL14
UL49 (ORF49)	594	594	594	Virion protein kinase	UL13
UL50 (ORF50)	508	508	508	Alkaline DNase	UL12
UL51 (ORF51)	74	74	74	Myristylated virion protein	UL11
UL52 (ORF52)	450	450	450	Glycoprotein M (gM)	UL10
UL53 (ORF53)	887	887	887	Origin-binding protein	UL9
UL54 (ORF54)	716	716	716	DNA helicase/primase complex	UL8
UL55 (ORF55)	303	303	303	Tegument protein	UL7
UL56 (ORF56)	753	753	753	Virion portal protein	UL6
UL57 (ORF57)	881	881	881	DNA helicase/primase complex	UL5
UL58 (ORF58)	225	225	225	Nuclear non-structural protein	UL4
UL59 (ORF59)	179	179	179	Unknown	–
UL60 (ORF60)	212	212	212	Colocalizes with ICP22	UL3
UL61 (ORF61)	312	312	301	Uracil–DNA glycosylase	UL2
UL62 (ORF62)	218	218	218	Envelope glycoprotein L (gL)	UL1
UL63 (ORF63)	532	419	419	Promiscuous transactivator (EICP0)	IE110
IR1 (ORF64)	1487	1487	1487	Transcriptional activator (IE)	IE175
IR2 (ORF77)	1165	1165	1165	Negative regulatory protein (IR2P)	–
IR3 (ORF78)	95	95	95	Anti-sense RNA to the IE mRNA	–
IR4 (ORF65)	293	293	293	Host range determinant; co-transactivator	US1
IR5 (ORF66)	236	236	236	Virion protein	US10
IR6 (ORF67)	272	272	272	Virulence factor	–
US1 (ORF68)	418	246	246	Virion protein	US2
US2 (ORF69)	382	382	382	Protein kinase	US3
US3 (ORF70)	411	411	411	Glycoprotein G (gG)	US4
US4 (ORF71)	797	797	379	Glycoprotein 2 (gp2), respiratory virulence	US5
US5 (ORF72)	452	452	452	Glycoprotein D (gD)	US6
US6 (ORF73)	424	424	–	Glycoprotein I (gI); cell-to-cell spread of the virus	US7
US7 (ORF74)	550	550	–	Glycoprotein E (gE); cell-to-cell spread of the virus	US8
US8 (ORF75)	130	130	–	“10 K”	–
US9 (ORF76)	219	219	219	Tegument protein; may play a role in axonal spread	US9

*^a^Data for Ab4 are from Telford et al. with permission (23). Data for RacL11 and KyA are based on genomic sequences reported in this paper (GenBank: MF975655 for KyA and GenBank: MF975656 for RacL11). Size of each gene in codons does not include the stop codon*.

*^b^Function is assigned primarily from published data for proteins of herpes simplex virus 1 (HSV-1) (23, 43, 44). The discovery and function of EHV-1 IR2 (25, 26), IR3 (27–29), UL1 and UL2 (39), UL3 (45), UL11 (46), UL20 (47), UL31 (48), UL34 (49), US4 (gp2) (50), and US6 (gI) and US7 (gE) (37) were reported previously*.

*–, not present*.

To assess the point mutations, the amino acid (aa) sequences of all ORFs were compared between the KyA and RacL11 strains (Table 2). Thirty six point mutations were detected in 14 different ORFs of the KyA genome. Fifty nine ORFs were completely conserved among the sequences of KyA and RacL11 examined. Interestingly, both the IR2 (ORF77) gene (25, 26) and the IR3 (ORF78) ORF (27–29) that were discovered to be regulatory genes in the KyA genome are conserved in both the RacL11 and Ab4 genomes.

**Table 2:** Amino acid substitutions in the Kentucky A (KyA) and RacL11 proteins compared to Ab4.

Protein	No.	Position^a^	RacL11	KyA	Ab4
UL5 (ORF5; multifunctional expression regulator)	1	8	R	S	S
	2	64	D	N	D
	3	87	I	S	S
	4	208	R	R	S
UL13 (ORF13; tegument protein VP13/14)	1	409	Y	S	Y
UL16 (ORF16; envelope glycoprotein C)	1	107	E	K	E
	2	145	K	K	E
	3	166	K	Q	Q
	4	275	V	A	V
UL18 (ORF18; DNA polymerase processivity subunit)	1	188	Q	P	Q
	2	320	N	N	T
UL21 (ORF21; ribonucleotide reductase subunit 1)	1	521	F	I	I
UL23 (ORF23; tegument protein UL37)	1	559	A	S	A
UL33 (ORF33; envelope glycoprotein B)	1	16	H	H	N
	2	132	A	D	A
	3	593	E	K	E
	4	734	A	V	V
	5	976	D	N	N
UL34 (ORF34; Protein V32)	1	52	Q	H	Q
	2	131	R	W	W
UL38 (ORF38; thymidine kinase)	1	247	R	H	H
UL39 (ORF39; envelope glycoprotein H)	1	38	T	N	T
	2	143	E	T	E
UL42 (ORF42; major capsid protein)	1	985	R	G	R
UL52 (ORF52; envelope glycoprotein M)	1	61	Y	F	Y
	2	260	S	R	S
	2	372	N	Y	Y
	3	389	M	V	V
UL53 (ORF53; DNA replication origin-binding helicase)	1	222	I	I	L
	2	582	N	D	N
	3	858	P	T	T
	4	873	S	R	S
UL54 (ORF54; helicase-primase subunit)	1	539	T	H	H
	2	540	R	V	V
	3	541	V	S	S
	4	558	E	G	E

*^a^Position is the amino acid location in the open reading frames*.

### DNA Sequence Analysis of the U_L_ Region

**Figure 2:**
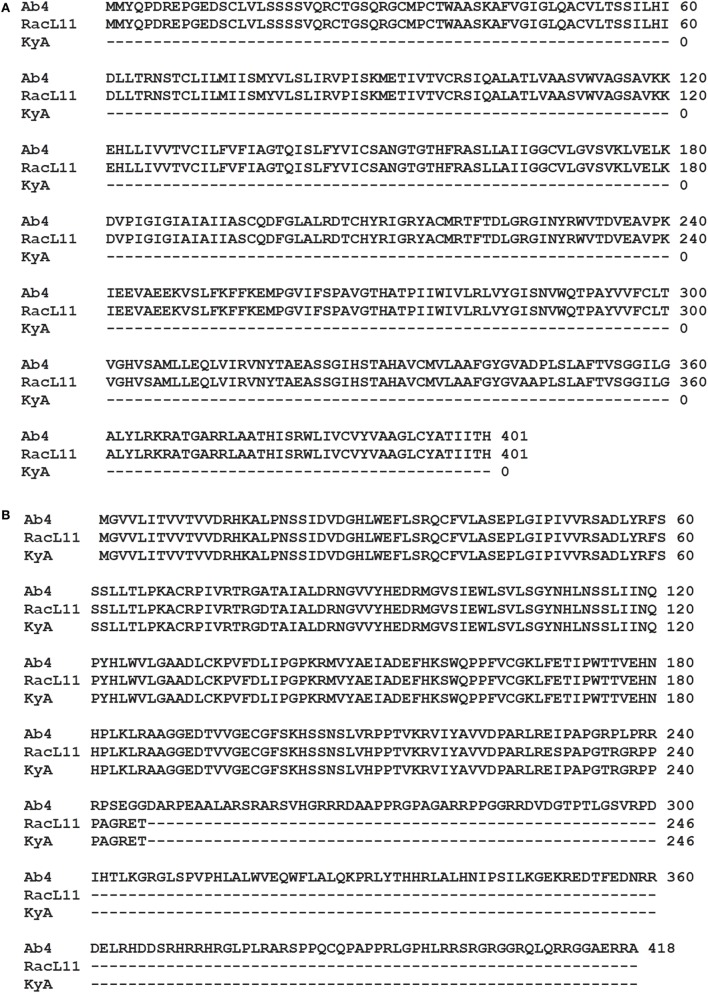
ORF17 and open reading frame (ORF) 68 in the Kentucky A (KyA) genome. **(A)** Predicted amino acid sequence of the ORF17 protein of RacL11 compared to that of Ab4 reveal perfect identity. Sequence analysis of the KyA genome revealed that ORF17 is deleted in KyA genome. **(B)** An identical in-frame deletion was detected in the US1 (OFR68) gene of the KyA and RacL11 genomes. Clustl W multiple sequence alignment software (http://www.ebi.ac.uk/Tools/msa/clustalw2/) was used for the alignment.

The EHV-1 genome is composed of a unique long (U_L_) region and a unique short segment (U_S_) that is flanked by two inverted repeats (IR and TR (21);). The U_L_ region of EHV-1 Ab4 is 112,935 bp that includes the 32 bp repeats. The sequencing results revealed that the U_L_ region of KyA totals to 109,697 bp that includes an identical 32 bp repeat at each end. The U_L_ region of RacL11 totals to 111,148 bp that also includes the identical 32 bp repeat at each terminus. In the KyA chromosome, the UL1 (ORF1), UL2 (ORF2), and UL17 (ORF17) genes are deleted as compared with the Ab4 sequence (Figures 1 and 2). These three major deletions in the U_L_ region of KyA have been reported (30, 31, 34) and were confirmed in this study. With regard to the RacL11 strain, the sequence of the ORF17 gene was identical to that of the Ab4 virus, and the ORF would encode a protein of 401 residues (Figure 2). Interestingly, the UL1 and UL2 genes were also deleted in the RacL11 strain. The deletion of these two ORFs in the RacL11 genome was surprising as the UL1 and UL2 genes were reported to be important for disease outcome and the modulation of cytokine responses (39).

ORF17 is non-essential for EHV-1 replication in cell culture (34). An identical in-frame deletion in the UL63 (ORF63; EICP0) gene of KyA and RacL11 genomes was observed (Figure 1 and Table 3). The results revealed that sequences encoding 113aa were deleted in the EICP0 gene of both strains. Our previous results showed that the KyA EICP0 protein functions as a potent transcriptional activator of all classes of EHV-1 promoters and, thus, the 113 residues (aa319–432) of the Ab4 EICP0 are not essential for its transactivation activities (35, 36). The sequencing results also showed that the addition of eight nucleotides AGCCGCCC at position 1,847 was detected in ORF14 of both KyA and RacL11 as compared with ORF14 of Ab4. Due to the addition of these nucleotides, the ORF14 of KyA and RacL11 was shifted such that the final length of the ORF14 protein is 626aa in contrast to the Ab4 ORF14 protein of 747aa.

**Table 3:** Deletions in the genomes and proteins of EHV-1 Kentucky A (KyA) and RacL11 as compared to equine herpesvirus 1 (EHV-1) Ab4.

Gene	Deletions of entire gene/open reading frame (ORF)		
	Ab4	RacL11	KyA
UL1: ORF1	No	Yes	Yes
UL2: ORF2	No	Yes	Yes
UL17: ORF17	No	No	Yes
US6: ORF73 (gI)	No	No	Yes
US7: ORF74 (gE)	No	No	Yes
US8: ORF75	No	No	Yes

**ORF**	**In-frame deletions in ORF**		
	**Ab4**	**RacL11**	**KyA**

UL14: ORF14	No	Yes (Δaa616–747)	Yes (Δaa616–747)
UL61: ORF61	No	No	Yes (Δaa148–301)^a^
UL63: ORF63 (EICP0)	No	Yes (Δaa319–432)	Yes (Δaa319–432)
US1: ORF68	No	Yes (Δaa235–237)	Yes (Δaa235–237)
US1: ORF68	No	Yes (Δaa243–418)	Yes (Δaa243–418)
US4: ORF71 (gp2)	No	No	Yes (Δaa75–482)

*^a^The ORF of the UL61 protein of KyA has a frame-shift at amino acid 148 such that the ORF of aa148–301 differs from that of the UL61 protein of RacL11 and Ab4*.

### DNA Sequence Analysis of the U_S_ Segment

The U_S_ segment of EHV-1 Ab4 is 11,861 bp long and is flanked with the IR and TR of 12,714 bp each (23). The DNA sequencing results showed that the KyA U_S_ segment is 6,759 bp flanked with IR and TR sequences of 12,446 bp each, while the U_S_ segment of RacL11 is 11,872 bp flanked with IR/TR of 12,223 bp each. EHV-1 KyA has a total of 268 bp deletions in each inverted repeat, located between ORF63 and ORF64. RacL11 has a total of 491 bp deletions in each inverted repeat, located upstream of ORF64 and upstream of ORF65. The genome of EHV-1 Ab4 does not have these deletions. DNA sequence analysis of the U_S_ segment of KyA revealed that genes US6 (ORF73; gI), US7 (ORF74; gE), and US8 (ORF75; 10 K) were deleted as compared to the sequences of Ab4 and RacL11 (Figure 1 and Table 3). An in-frame deletion in the KyA US4 (gp2) gene was detected. An identical in-frame deletion in the US1 (OFR68) gene of the KyA and RacL11 genomes was also detected, and this deletion would result in the ORF68 protein of KyA and RacL11 being 246aa in contrast to the 418aa ORF68 protein of Ab4 (Figure 2B). Whether the difference in the amino acid sequence of the carboxyl terminus of the KyA and RacL11 ORF68 protein affects the function of this virion protein remains to be determined. EHV-1 glycoproteins gI, gE, and full-length gp2 are involved in cell-to-cell spread and contribute to the pathogenesis of EHV-1 (37, 38, 50). These results suggest that the deletion of gI, gE, and gp2 in KyA has important roles in the attenuation of this EHV-1 strain.

### Growth Kinetics of KyA, RacL11, and Ab4 in Cell Culture

**Figure 3:**
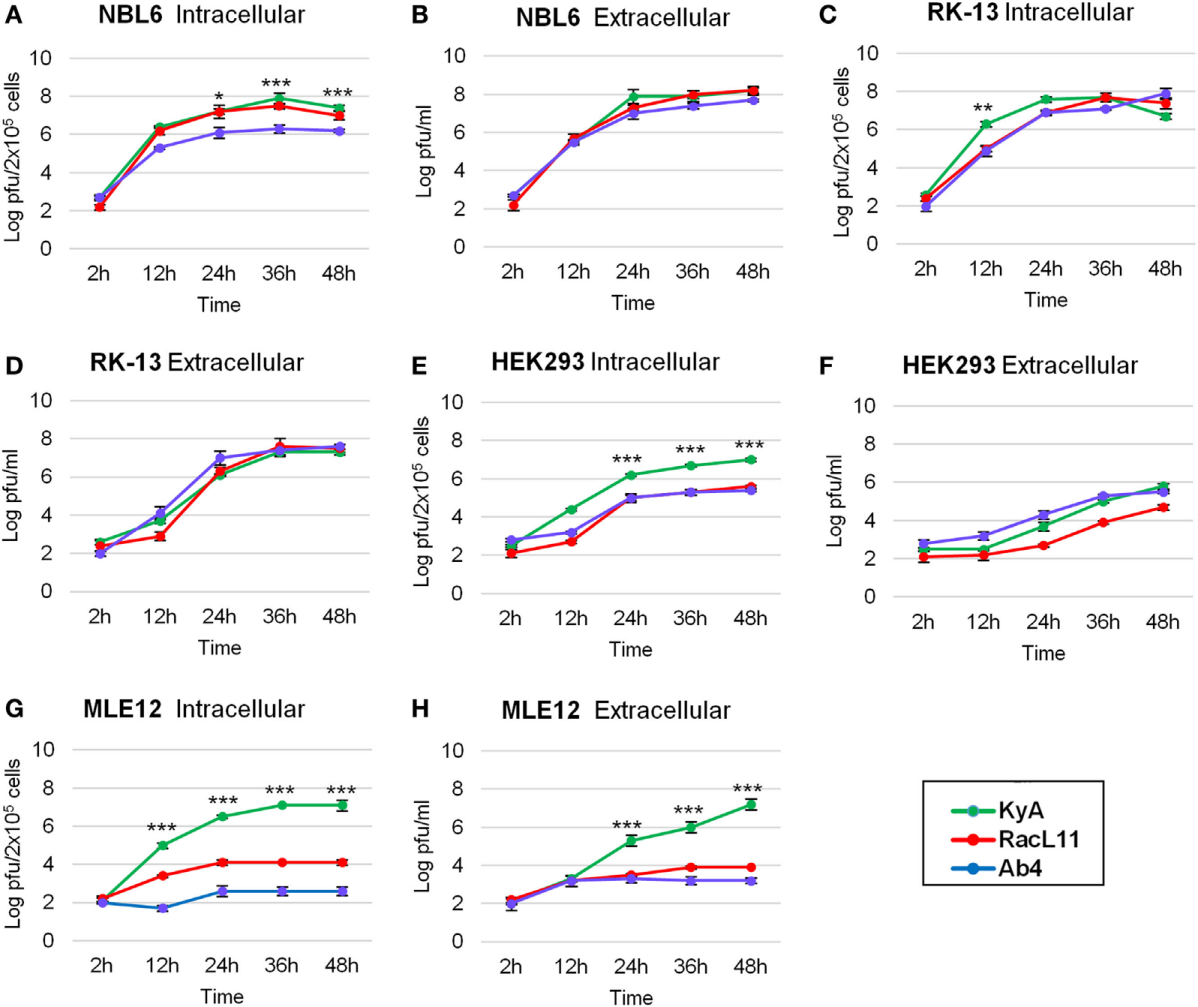
Growth kinetics of equine herpesvirus 1 (EHV-1) strains in four cell types. Intracellular and extracellular growth kinetics of EHV-1 Kentucky A (KyA), RacL11, and Ab4 in equine NBL6 cells **(A,B)**, rabbit RK-13 cells **(C,D)**, human HEK293 cells **(E,F)**, and mouse MLE12 cells **(G,H)**. Cell monolayers were infected at a multiplicity of infection of 0.05. Culture medium and cells were harvested at 2, 12, 24, 36, and 48 h post-infection. The intracellular virus was released by three freeze–thaw cycles. Virus titer was determined by plaque assay on NBL6 cells. Data are averages and are representative of three independent experiments. Error bars indicate SD. Statistical differences between KyA and RacL11 (or Ab4) viruses were labeled (*P < 0.05, **P < 0.01, and ***P < 0.001).

One-step growth experiments of the three EHV-1 strains, KyA, RacL11, and Ab4, were conducted to examine whether mutations detected in KyA affect viral growth in mouse MLE12 rabbit RK-13, equine NBL6, and human HEK293 cells. The monolayers were infected with each of the three EHV-1 strains at a MOI of 0.05 and were harvested at 0, 12, 24, 36, and 48 h post-infection (hpi). Culture medium was also collected at the same time points. Extracellular and intracellular virus titers were determined by plaque assay on NBL6 cells. KyA and RacL11 exhibited identical growth kinetics in NBL6 cells (Figures 3A,B). Whereas the titer of Ab4 was 2 logs lower than those of KyA and RacL11 (Figure 3A). In RK-13 cells, the growth patterns of the RacL11 and Ab4 strains were similar, although the intracellular titer of KyA was higher than those of the RacL11 and Ab4 strains (Figures 3C,D). In HEK293 cells, the RacL11 and Ab4 showed similar growth patterns (Figures 3E,F). By contrast, the intracellular titer of KyA was significantly higher at all time points (Figure 3E). In MLE12 cells, the extracellular and intracellular titers of KyA significantly increased rapidly with time while Ab4 was not able to grow in these mouse cells (Figures 3G,H). Overall, these results demonstrated that deletions and point mutations in the KyA genome presented in this study did not impair KyA growth or tropism for any of the four cell types.

### Pathogenicity of EHV-1 KyA, RacL11, and Ab4 strains in CBA Mice

**Figure 4:**
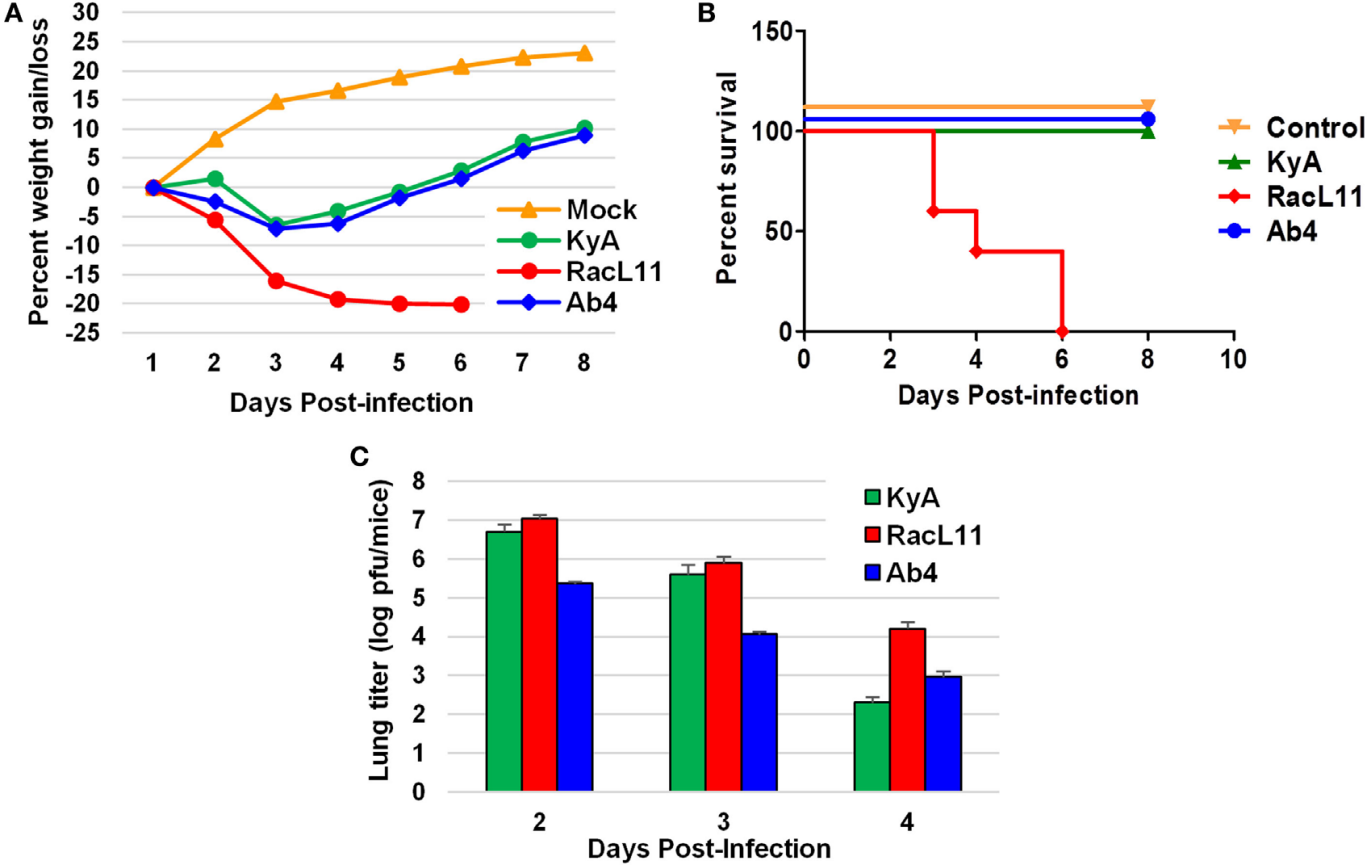
Pathogenicity of equine herpesvirus 1 (EHV-1) Kentucky A (KyA), RacL11, and Ab4 in CBA mice. Mice were infected intranasally with 1.5 × 10^6^ plaque forming unit of KyA, RacL11, or Ab4. Cell culture medium was used for mock infection. **(A)** Mice (*n* = 5 per group) were weighed daily at days 1–8. The body weights were expressed in average percentage gain or loss relative to the initial body weight. **(B)** Percentage survival at days post-infection in mock- or virus-infected mice. **(C)** Virus titers of lungs of EHV-1-infected CBA mice. At days 2, 3, and 4 pi, lungs were removed and homogenized, and the amounts of KyA, RacL11, and Ab4 were determined by standard plaque titration on NBL6 monolayers.

CBA mice were infected intranasally with KyA, RacL11, or Ab4 (1.5 × 10^6^ PFU/mouse) and were monitored daily for weight gain or loss and morbidity. Mouse lungs were harvested and homogenized following EHV-1 infection, and the amount of infectious virus was determined. RacL11-infected mice lost more than 20% of their pre-infection body weight by day 4 pi and succumbed by 3 to 6 dpi (Figures 4A,B). By contrast, KyA- and Ab4-infected mice lost less than 10% of their pre-infection body weight and rapidly regained their weight. None of the mice succumbed to KyA infection (Figures 4A,B). Virus titers in the lungs of KyA-infected mice were 100-fold lower at 4 dpi than those of the lungs of RacL11-infected mice (Figure 4C). These results demonstrate that EHV-1 KyA and Ab4 are apathogenic in the CBA mouse model and are consistent with previous studies (14, 37, 38, 50, 51).

### Phylogenetic Analysis

**Figure 5:**
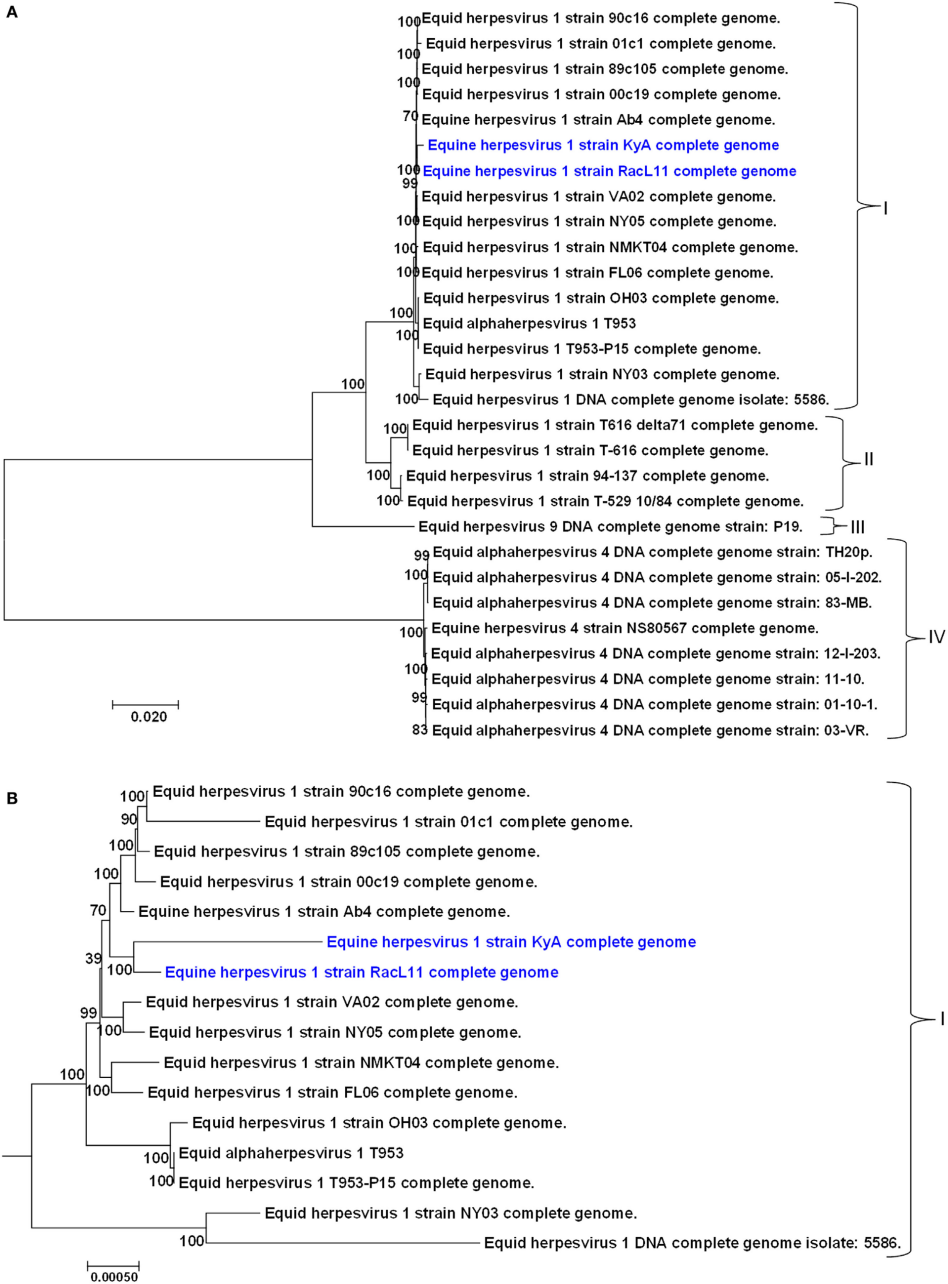
Phylogenetic analyses of the complete genomes of strains of equine herpesvirus 1 (EHV-1), EHV-4, and EHV-9. **(A)** The phylogenetic tree indicating evolutionary relationships was generated from the genomic sequence alignments of the Kentucky A (KyA) (GenBank: MF975655), RacL11 (GenBank: MF975656), and 27 other EHV strains using Molecular Evolutionary Genetic Analysis software (41). **(B)** Subtree of group I reveals EHV-1 KyA and RacL11 genomes are closely related but the KyA genome exhibits a greater evolutionary distance. Bootstrap values out of 1,000 replicates are indicated as a percentage to the left of each branch of the tree. The bar at the bottom of the figure provides a scale that represents the amount of genetic change. The units of branch length are nucleotide substitutions per site divided by the length of the genomic sequence.

A phylogenetic tree was constructed to compare the genomes of KyA and RacL11 with the genomes of 27 other equine herpesviruses, including EHV-1, EHV-4, and EHV-9 (Figure 5). In the phylogenetic analysis, the 20 EHV-1 strains fell into two genetic groups. Group I consisted of 16 strains, while group II consisted of 4 strains. The KyA and RacL11 strains belonged to group I, and the analyses revealed that strains KyA and RacL11 are closely related to strains Ab4, 00c19, 89c105, 01c1, 90c16, VA02, NY05, NMKT04, and FL06. The eight strains of equine herpesvirus 4 were closely related and separated into a single group, group IV. Lastly, the sole strain of equine herpesvirus 9 fell into a separate group, designated as group III. Figure 5B presents the genetic relationships among EHV-1 strain of group 1 and reveals that KyA has a greater number of genetic changes as compared to RacL11 and Ab4.

## Discussion

It is noteworthy that the present study reveals the whole-genome sequence of attenuated EHV-1 KyA and wild-type (wt) RacL11 strains, both of which show several gene alterations as compared to the genome of wt EHV-1 Ab4 (Tables 2 and 3, Figures 1 and 2; KyA and RacL11 genomes GenBank MF975655 and MF975656, respectively). The sequencing results of the U_L_ region of KyA revealed genomic deletions of UL1 (ORF1), UL2 (ORF2), and UL17 (ORF17). Our previous sequencing results of portions of the KyA genome showed genomic deletions of UL1, UL2, UL17, US6 (ORF73; gI), US7 (ORF74; gE), and US8 (ORF75) and in-frame deletions in genes US1 (ORF68), US4 (ORF71; gp2), and UL63 (ORF63; EICP0) as compared to the sequences of Ab4 (30–35). The roles of the UL1 and UL2 in EHV-1 have been investigated (39), and the data showed that deletion of these two genes from the EHV-1 strain Ab4 attenuated disease despite the fact that both wt and mutant viruses showed similar *in vitro* growth kinetics. These results suggest that the deletion of the UL1 and UL2 genes is involved in the attenuation of the KyA strain.

We confirmed an identical in-frame deletion in the EICP0 gene in the genomes of KyA and RacL11 such that the EICP0 regulatory protein of these two strains would be 419 amino acids in contrast to the 532-amino acid protein of Ab4. The demonstration that the EICP0 protein of KyA has potent transactivation activity of all classes of EHV-1 promoters (35, 36) indicates that residues 319–432 of the Ab4 EICP0 protein are not essential for its transactivation activity. Our sequence analyses of the U_S_ segment showed genomic deletions of gI, gE, 10 K, and gp2 in KyA as compared to the Us of RacL11 and Ab4. The role of gI and gE in viral pathogenesis is well established. Previously, it was demonstrated that the restoration of gI and gE to attenuated KyA generated the *KgI/gE* virus that exhibited a virulence phenotype in the CBA mouse (38). The *KgI/gE* virus was able to spread to the brains of CBA mice after intranasal infection, resulting in meningoencephalitis (52), which is consistent with the role of gI and gE observed in EHV-1-infected horses exhibiting neurological signs (9). However, the deletion of glycoproteins gI and gE from wt RacL11 did not significantly reduce clinical signs of respiratory disease in mice (38), suggesting that multiple viral genes are involved in the virulence phenotype. The deletion of gene US4 (gp2) in EHV-1 Ab4 resulted in its attenuation, and the generated virus conferred protection against pulmonary disease in mice after challenge with wt virus (16). The insertion and expression of the full-length RacL11 gp2 (791aa) in KyA generated a recombinant virus that elicited fatal immunopathological responses in the lower respiratory tract of CBA mice, indicating a role of US4 in EHV-1 pathogenesis (50). Overall, we conclude that the six genomic deletions UL1, UL2, UL17, gI, gE, and US8 and two in-frame deletions in ECIP0 and gp2 may contribute to the attenuation of EHV-1 KyA.

The data presented in this study revealed that the KyA strain replicated to titers that exceeded those observed for strains RacL11 and Ab4 in all four cell types examined. The growth kinetics results revealed that mutations in KyA have no inhibitory effect on its replication, and the rapid replication of KyA to high titers makes this strain an attractive vaccine candidate. Collaborative studies with Japanese researchers demonstrated that the Δ*gE*Δ*gI-lacZ* mutant of a pathogenic strain of EHV-1 replicated to high titers in cell culture, indicating that gE and gI are not required for viral growth *in vitro* (37). In another study, it was shown that deletion of UL1 and UL2 in EHV-1 Ab4 did not alter *in vitro* growth kinetics (39).

Our findings showed that all mice infected with RacL11 succumbed by 6 dpi while the KyA- or Ab4-infected mice survived. KyA and RacL11 were able to replicate in the lungs of CBA mice while Ab4 did not (Figure 3C). EHV-1 Ab4 is pathogenic in foals (39, 53) and is not pathogenic in mice because this strain is not mouse-adapted and cannot replicate in the lungs of mice. However, if the infectious dose of Ab4 was increased 10-fold to 1.5 × 10^7^ PFU, the CBA mice lost more body weight and 20% of the animals succumbed to infection by 6 dpi (data not shown). When infected with high dose (4 × 10^7^) of KyA, all the mice survived and showed weight loss/gain patterns similar to those of mock-infected mice (19). The observation that KyA and Ab4 are apathogenic in mice was also shown by van Woensel et al. (51). These results are consistent with our previously published findings that KyA is non-pathogenic in CBA mice and horses, whereas the wt RacL11 induces severe inflammatory cell infiltration in the lung, such that infected mice succumb at 4–6 dpi (14, 20, 38, 50).

In the phylogenetic study, KyA, RacL11, and Ab4 strains belong to group I (Figure 5). The group I consists of 16 EHV-1 strains including 11 EHV-1 strains sequenced in Japan and two strains T953 and T953-P15 sequenced in USA. The phylogenetic tree showed that both EHV-1 KyA and RacL11 are closely related to the Ab4 and Japanese strains.

## Conclusion

The findings of the present study reveal several genomic deletions and in-frame mutations in the KyA genome as compared to those of RacL11 and Ab4. *In vivo* studies demonstrated that KyA-infected mice lost less than 10% of their body weight, rapidly regained weight, and all mice survived. By contrast, the RacL11 strain induced severe infection, as evident by significant weight loss, ruffled fur, severe inflammation of the lungs, and an increased mortality rate. Our previous results showed that the immunization of CBA mice with KyA protected animals from wt RacL11 challenge (14, 37, 38, 50). *In vitro* results demonstrated that KyA was able to grow rapidly in a variety of cell types, including mouse, rabbit, equine, and human cells. The attenuation of KyA in the mouse model and the ability of KyA to replicate to high titers make it an attractive vaccine candidate. We are planning to study EHV-1 KyA in horses. Our future study will give more insight into the suitability of KyA as a vaccine to combat EHV-1-mediated disease in the horse. Understanding the role in attenuation of genomic deletions and mutations may help in the development of a safe and effective EHV-1 vaccine.

## Ethics Statement

All the animal experiments were done in accordance to the guidelines of the Louisiana State University Health Sciences Center at Shreveport Institutional Animal Care and Use Committee (IACUC). The LSUHSC-Shreveport OLAW assurance number is A3095-01. Ethical clearance for all animal studies was taken from the LSUHSC-Shreveport IACUC under protocols P-16-030 and P-15-038.

## Author Contributions

AS performed most of the experiments, analyzed data, and drafted the manuscript. DO conceived of the study, analyzed data, and drafted the manuscript. SK conceived of the study, performed some of the experiments, analyzed data, and drafted the manuscript. All authors read and approved the final manuscript.

## Conflict of Interest Statement

The authors declare that the research was conducted in the absence of any commercial or financial relationships that could be construed as a potential conflict of interest.
